# Statewide evaluation of infection control measures for preventing coronavirus disease 2019 in hemodialysis facilities

**DOI:** 10.6061/clinics/2021/e3299

**Published:** 2021-09-28

**Authors:** Ana Rubia Guedes, Bruno de Melo Tavares, Denise Brandão de Assis, Maristela Pinheiro Freire, Geraldine Madalosso, Anna S. Levin, Lauro Vieira Perdigão, Maura Salaroli de Oliveira

**Affiliations:** I Departamento de Controle de Infeccao, Hospital das Clinicas HCFMUSP, Faculdade de Medicina, Universidade de Sao Paulo, Sao Paulo, SP, BR.; II Divisao de Infeccoes Hospitares, Centro de Vigilancia Epidemiologica “Prof. Alexandre Vranjac”, Centro de Controle de Doencas, Secretaria de Estado da Saude, Sao Paulo, SP, BR.; III Departamento de Doencas Infecciosas, Laboratorio de Investigacao Medica - LIM 49 e Instituto de Medicina Tropical, Universidade de Sao Paulo, Sao Paulo, SP, BR.

**Keywords:** Hemodialysis, COVID-19, Cluster, Statewide Survey, Infection Control

## Abstract

**OBJECTIVE:**

This study aimed to evaluate the occurrence of coronavirus disease 2019 (COVID-19) in hemodialysis facilities and the occurrence of and risk factors for clustering of COVID-19 cases.

**METHODS:**

We conducted a cross-sectional online survey between March and July 2020, in all dialysis facilities in São Paulo state, using Google Forms. The online questionnaire contained questions addressing specific components of infection prevention and control practices and the number of cases during the COVID-19 pandemic.

**RESULTS:**

A total of 1,093 (5%) COVID-19 cases were reported among 20,984 patients; approximately 56% of the facilities had ≥1 cluster. Most facilities implemented various measures (such as allocation of dedicated COVID-19 areas/shifts, symptom screening, environmental disinfection, and maintenance of adequate ventilation) to prevent the transmission of severe acute respiratory syndrome coronavirus 2. Clustering of COVID-19 cases was suspected in only 7% of dialysis facilities. The only variable associated with this event was the performance of aerosol-generating procedures (odds ratio: 4.74; 95% confidence interval: 1.75-12.86).

**CONCLUSION:**

Attention should be paid to avoiding the performance of aerosol-generating procedures in dialysis facilities and monitoring the clustering of cases.

## INTRODUCTION

The coronavirus disease 2019 (COVID-19) pandemic has affected more than 170 million people, with more than 3 million deaths reported as of May 2021 (1). Besides its occurrence in the community, COVID-19 can also be acquired through exposure to healthcare environments, such as hemodialysis (HD) centers, with confirmed or suspected COVID-19 patients. Approximately 2-3 million patients are undergoing dialysis worldwide and are continuously exposed to the healthcare facilities and healthcare workers (HCWs). Furthermore, they traveled to and from dialysis facilities during the pandemic, even during periods of high virus circulation (2). Patients in dialysis facilities have more than two-fold the risk of acquiring COVID-19 than those undergoing dialysis at home (3).

In addition to having a higher risk of severe acute respiratory syndrome coronavirus 2 (SARS-CoV-2) infection, patients on HD have worse COVID-19 outcomes. They frequently have comorbidities that are associated with worse prognosis, such as diabetes mellitus, hypertension, and lung disease, and are considered immunocompromised (4). HD patients were more likely to be hospitalized owing to COVID-19 and to have a longer hospital stay. Furthermore, COVID-19 mortality rates of patients on HD vary from 8% to 31%, at least five times higher than those in the general population (3).

Therefore, implementation of effective strategies to prevent COVID-19 transmission in this high-risk population is of utmost importance. Dedicating areas and shifts for COVID-19 suspected/confirmed cases, screening respiratory signs and symptoms, reinforcing environmental cleaning, and implementing droplets/aerosol and contact precautions are relevant measures that must be assured (1).

To date, studies evaluating the safety of patients against COVID-19 in HD facilities are limited and their findings are not conclusive. A more detailed investigation of HD centers is warranted to provide relevant information and enhance the understanding on the risk of infection among dialysis patients. The findings of this investigation will serve as a basis for developing public health interventions to minimize the rates of morbidity and mortality caused by COVID-19. This study aimed to evaluate the incidence of COVID-19 among patients in HD facilities and the incidence of COVID-19 clusters in these facilities as well as the associated factors.

## METHODS

### Setting, patients, and study design

The state of São Paulo, the most populous Brazilian state, has 45 million inhabitants. It has 645 cities and 198 dialysis facilities. Since 2015, there has been a statewide surveillance system coordinated by the São Paulo State Health Department to monitor healthcare-associated infections in these facilities. We conducted a survey to evaluate the incidence of COVID-19 among patients and HCWs and to evaluate infection control measures implemented in dialysis facilities.

The online questionnaire contained questions addressing specific components of infection prevention and control practices and the occurrence of cases during the COVID-19 pandemic. The questions were sent out to all dialysis facilities in São Paulo state using Google Forms. We requested the person in charge of infection prevention in the facility to respond to the survey, and three follow-up emails were sent to encourage responses. The facilities also reported data on patients and HCWs with confirmed and suspected COVID-19: date of symptom onset, dialysis shift, previous contact with COVID-19 at home and at the facility, and diagnostic method employed. No financial incentives were provided to those individuals who participated in this study. The study was conducted from March 1 to July 31, 2020.

### Definitions

- Suspected COVID-19: Any person with at least one of the following symptoms: cough; fever; shortness of breath; sudden onset of anosmia, ageusia, or dysgeusia
- Confirmed COVID-19: Any person with a positive RT-PCR test result for SARS-CoV-2 in a clinical specimen, a positive serological result, or radiological lesions compatible with COVID-19 (e.g., bilateral or peripheral ground-glass opacities)
- Healthcare worker: Any professional working within a dialysis facility
- Cluster: Occurrence of more than one confirmed COVID-19 case within 7 days (linked by time) during the same dialysis shift (linked by location)

### Data analysis

Initially, a descriptive analysis was conducted. We then compared facilities that had at least one COVID-19 cluster with facilities with no clusters, including only those that sent complete data and performed RT-PCR tests for the detection of SARS-CoV-2. Continuous variables were compared using Student’s t-test (for normally distributed variables) or the Mann-Whitney U test (for non-normally distributed variables). Categorical variables were evaluated with the χ^2^ test or two-tailed Fisher’s exact test, as appropriate, using an EpiInfo software (version 7.0; CDC, Atlanta, USA). The odds ratios and 95% confidence intervals (CIs) were calculated. All p-values were two-tailed, and a *p*-value ≤0.05 was considered significant.

The Institutional Ethics Committee in Research approved this study (number: 032597183.0000.0068). Informed consent was obtained electronically.

## RESULTS

The survey questionnaire was sent to all 198 dialysis facilities in the state, located in 73 cities. A total of 121 facilities (61%) responded to the survey. In 100 (83%) facilities, the nurse was assigned to answer the survey; in another 73 (60%) facilities, the person in charge of managing the facility was assigned to answer the survey. These facilities managed a total of 20,984 patients and had 4,333 dialysis machines.

Only 3 of the 121 facilities had individual patient rooms or boxes. The other facilities had a large hall with several armchairs with a minimum distance of 1 m between them (in accordance with the Brazilian law). The characteristics of these facilities are listed in Table 1.

The provision of an RT-PCR test for SARS-CoV-2 testing of patients and HCWs was available in 110 (91%) and 96 (79%) facilities, respectively. During the study period, a total of 2,024 patients were suspected of having COVID-19, of whom 1,093 (5%) were confirmed of having the disease. Among the confirmed patients, 244 (22%) had close contacts with a household member with COVID-19, whereas 195 (18%) had close contacts with HCWs with COVID-19 in the dialysis facility.

A total of 1,115 HCWs were suspected of having COVID-19, of whom 459 (40%) were confirmed of having the disease. Among the confirmed HCWs, 397 (86%) were professionals involved in providing direct patient assistance. Moreover, 114 (25%) had close contacts with a household member with COVID-19, whereas 256 (56%) had close contacts with HCWs with COVID-19 in the dialysis facility.

The infection prevention measures adopted in the facilities are listed in Table 1. Most facilities had areas and/or shifts allotted for patients with either suspected or confirmed COVID-19 and implemented patient and HCW screening for COVID-19-related symptoms. Approximately 31% of the facilities reported difficulty in acquiring personal protective equipment. Meanwhile, 31 (26%) facilities reported performing some type of aerosol-generating procedures (AGPs) (*e.g.*, intubation, oxygen therapy/inhalation, and collection of diagnostic respiratory specimens) in the patient area.

Although only nine (7%) facilities reported having a COVID-19 cluster, based on our definition, 61 (56%) of the 108 facilities that provided complete data and offered RT-PCR diagnostic testing had at least one cluster. A total of 181 clusters were reported (median: 2 patients/cluster; range: 2-17). These clusters were responsible for the occurrence of 447 COVID-19 cases (41% of confirmed COVID-19 cases). The performance of AGPs was the only factor associated with COVID-19 clusters (odds ratio: 4.74) (Table 1).

## DISCUSSION

We evaluated the data of 20,984 adult patients treated at 121 hemodialysis facilities. Approximately 5% of patients were diagnosed with COVID-19 in the first 5 months of the pandemic. In June 2020, the city of São Paulo reported a seroprevalence of 9.5% (5). Despite this relatively low proportion of confirmed cases, 56% of the facilities had clusters; thus, they may have had healthcare-associated transmission. The risk factor associated with clusters of COVID-19 was the performance of AGP in patient areas.

Several recommendations for the control of COVID-19 in outpatient dialysis facilities have been published (6), but only limited data on the adherence to infection control practices in HD facilities are available. Our study was conducted at the beginning of the pandemic, a period when there was conflicting information on whether COVID-19 is spread through aerosol or droplet transmission. We believe that this uncertainty is reflected in the mask policy adopted in HD facilities: half of the facilities adopted the use of N95 masks, whereas half adopted the use of surgical masks. Opening of windows in the patient care area to improve indoor air quality is a common practice, although this practice contradicts the current recommendations on ventilation systems in critical areas (7). However, the presence of clusters was not associated with the level of adherence to any of the recommended control measures: allotment of COVID-19 areas or shifts, screening of symptoms presented by HCW and patients, environmental cleaning and disinfection, or ventilation of the facility (6).

The only factor associated with having clusters was the performance of AGPs in the facility. The relative importance of aerosol transmission of SARS-CoV-2 remains incompletely understood, and the definition of what constitutes an AGP is still being reassessed (8). Klompas et al. suggested that four factors might explain the risk of transmission during the performance of medical procedures: existence of forced air into airways, presence of symptoms and disease severity of the source patient, distance of the source, and duration of exposure to aerosols (9). Our findings suggest an association between the occurrence of clusters and the performance of AGPs. We used a rather ample definition of AGP, including the collection of respiratory samples, intubation, and administration of oxygen therapy. Therefore, procedures performed in the upper respiratory tract pose a risk of COVID-19 transmission in HD facilities. As there remains no definitive and comprehensive list of AGPs that are commonly performed in healthcare settings (10), we believe that our findings contribute toward understanding the factors that lead to the increased transmission risk.

An important point is that only 7% of the facilities recognized the occurrence of clusters, which may have delayed the implementation of corrective measures. Because of this, most clusters were not investigated as such, potentially increasing the extent of transmission in the facility. This may explain why almost half of the patients with confirmed COVID-19 were considered part of the cluster. In addition, it is worrisome that 12% of facilities adopted incorrect isolation precautions, placing the HCWs at risk. These findings suggest that education and training on the basic concepts of hospital epidemiology should be a priority of the government and part of facility efforts toward improving the preparedness of dialysis facilities in facing epidemics. Most facilities had access to molecular testing for SARS-CoV-2, even in a middle-income country such as Brazil. This is an essential tool for prompt identification and isolation. However, this tool can only be useful if clusters are suspected.

Our study has some limitations. First, it was not possible to evaluate the exact rate of implementation of measures for preventing COVID-19, as it was self-reported, and facility visits were not conducted. Second, a response rate of 61% may affect the generalizability of our results. Third, it was not possible to study the occurrence of COVID-19 among HCWs owing to lack of data. Finally, the different methods to confirm the cross-transmission of SARS-CoV-2 in facilities, such as sequencing, were not employed.

In conclusion, our statewide survey demonstrated that most HD facilities implemented measures to prevent SARS-CoV-2 transmission. However, transmission may have occurred in over half of the facilities. The only variable associated with COVID-19 clusters was the performance of AGPs in patient areas.

## AUTHOR CONTRIBUTIONS

Guedes AR provided substantial contributions to the study conception and design, data collection and analysis, and was also responsible for the manuscript final version approval. Assis DB and Madalosso G contributed to the study design, and manuscript final version approval. Tavares BM and Freire MP contributed to the interpretation of data, and manuscript drafting and final version approval. Levin AS contributed to the study design, data interpretation, and manuscript drafting and final version approval. Perdigão Neto LV contributed to the study design, and manuscript drafting and final version approval. Oliveira MS provided substantial contributions to the study conception and design, data collection and interpretation, and manuscript drafting and final version approval.

## Figures and Tables

**Table 1:** Structural characteristics of and infection prevention measures adopted in hemodialysis facilities of the state of São Paulo during the COVID-19 pandemic and bivariate analysis of variables associated with the occurrence of COVID-19 clusters (March to July 2020).

Variable	Total number of hemodialysis facilities N:121	Occurrence of clusters* N:108		*p*	Odds ratio (95% CI)
		Yes n=61 (%)	No n=47(%)		
Administration					0.72
Private for profit	80 (66)	9 (50)	9 (50)		
Private non-profit	22 (18)	41 (57)	31 (43)		
Public	19 (16)	11 (61)	7 (39)		
Located within a hospital	61 (50)	26 (49)	27 (51)	1.81 (0.84-3.92)	0.13
Infection prevention measures					
Has an area allotted for patients with suspected/confirmed COVID-19	107 (88)	56 (58)	40 (42)	0.51 (0.15-1.72)	0.43
Has a shift allotted for patients with suspected/confirmed COVID-19	102 (84)	50 (56)	39 (44)	1.07 (0.39-2.92)	0.89
Screens every patient entering the facility for signs and symptoms of COVID-19	110 (91)	57 (58)	41 (42)	0.48 (0.13-1.80)	0.33
Screens every HCW entering the facility for signs and symptoms of COVID-19	78 (64)	38 (53)	34 (47)	1.58 (0.70-3.60)	0.27
Opens windows in the patient care area	82 (68)	41 (56)	32 (44)	1.04 (0.46-2.35)	0.92
Keeps air conditioning on	80 (66)	41 (56)	32 (44)	1.04 (0.46-2.35)	0.92
Performs hand hygiene audits	104 (86)	52 (56)	41 (44)	1.18 (0.34-4.38)	0.77
Precautions used to avoid COVID-19 transmission					
Contact and droplets/aerosols	108 (88)	57 (58)	39 (42)	2.92 (0.82-10.38)	0.12
Droplets and aerosols	9 (8)				
Contact	2 (2)				
Standard precautions	2 (2)				
Type of mask used by administrative HCW					
Surgical mask	114 (94)	58 (57)	44 (43)	0.76 (0.10-5.96)	0.74
Cloth mask	7 (6)				
Provides masks to patients with COVID-19	117 (97)	60 (58)	44 (42)	0.24 (0.02-2.42)	0.32
Provides masks to all patients	100 (83)	51 (57)	38 (43)	0.83 (0.31-2.24)	0.71
Performs aerosol-generating procedures in the patient area	31	25 (81)	6 (19)	4.74 (1.75-12.86)	0.001
Collection of diagnostic respiratory specimens	15 (13)				
Intubation	11 (9)				
Oxygen therapy/inhalation	5 (4)				
Reported the occurrence of a COVID-19 cluster	9 (7)	53 (54)	46 (46)	0.15 (0.003-1.16)	0.07

* 108 facilities that sent all data regarding the presence of COVID-19 cases and implemented measures. HCW, healthcare workers.
